# Hepatic Brucelloma Diagnosis and Long-Term Treatment, France

**DOI:** 10.3201/eid2505.180613

**Published:** 2019-05

**Authors:** Marie Amsilli, Olivier Epaulard, Jean-Paul Brion, Patricia Pavese, Christian Letoublon, Isabelle Pelloux, Max Maurin

**Affiliations:** Centre Hospitalier Universitaire de Grenoble, Grenoble, France (M. Amsilli, O. Epaulard, J.-P. Brion, P. Pavese, C. Letoublon, I. Pelloux, M. Maurin);; Université Grenoble Alpes, Grenoble (O. Epaulard, C. Letoublon, M. Maurin)

**Keywords:** brucellosis, hepatic brucelloma, Brucella, bacteria, liver abscess, molecular biology, antimicrobial therapy, abscess drainage, complications, zoonoses, France

## Abstract

We report a case of hepatic brucelloma in France. This diagnosis may be suspected in any patient who has a liver abscess after traveling to a brucellosis-endemic area. *Brucella* spp. may be detected by PCR in the liver tissue or suppuration. Abscess drainage and prolonged antimicrobial therapy help achieve healing.

Brucellosis is a zoonosis found worldwide (*1*,*2*) caused by gram-negative, facultative intracellular bacteria of the genus *Brucella.* Approximately 500,000 new infections are diagnosed annually, mainly in the Mediterranean basin, the Middle East, Latin America, and Asia (*1*–*3*). Brucellosis is a rare and mainly imported disease in other countries, including France (*1*,*4*). *Brucella* infection usually occurs after contact with infected animals or consumption of contaminated unpasteurized dairy products. Hepatic brucelloma (HB) is a chronic form of brucellosis arising up to 40 years after initial infection (*1*,*3*,*5*). Only 60 cases (1%–2% of all brucellosis infections) have been reported in English-language literature since 1904 (*1*,*3*,*5*,*6*). HB is associated with nonspecific systemic clinical symptoms (e.g., fever, malaise, weight loss, upper abdominal pain), moderate biologic abnormalities, and typical hypodense hepatic lesion with peripheral enhancement and central calcification (*1*–*3*,*5*,*6*). 

In April 2015, a previously healthy 55-year-old woman was referred to Grenoble University Hospital after 7 days of fever, asthenia, and weight loss. She had lived in France for 20 years, but had been born in and had traveled every year to Algeria. Her clinical examination was unrevealing. Blood tests showed moderate inflammation and anicteric cholestasis (Table). Hepatic ultrasound (HUS) and computed tomography (CT) confirmed a defect 60 mm in diameter in liver segments IV and VIII with several subcapsular liquid collections and central calcification (Appendix Figure, panel A). 

**Table:** Diagnostic and blood tests on patient with hepatic brucellosis, Grenoble, France

Test	Reference value	2015 Apr	2015 Nov	2016 Jan
Rose Bengal plate agglutination test (bioMérieux, https://www.biomerieux.com)	NA	Strongly positive	Positive	Negative
Tube agglutination test (Elitech, https://www.elitechgroup.com)	>80	160	20	<20
Immunofluorescence IgM (BioRad, http://www.bio-rad.com)	>40	<20	<20	<20
Immunofluorescence IgG (BioRad)	>80	160	160	320
*Brucella* PCR testing of liver tissue biopsies	NA	Positive for 16S rDNA	NA	Positive for *bcsp31* gene
Blood cultures	NA	Negative	Negative	Negative
Liver abscess and tissue cultures† (bacteria, fungi and mycobacteria)	NA	Negative	NA	Negative
C-reactive protein, mg/L	<3	56	5	4
Fibrinogen, g/L	2.1–4.1	6.1		
Leukocyte count, G/L	4–11	8.5	5	4.9
Neutrophil count, G/L	1.6–8.8	5.9	2.5	2.1
Thrombocyte count, G/L	150–400	489	287	284
Bilirubin, µmol/L	3–17	5	5	5
Alkaline phosphatase, IU/L	50–136	158	85	69
γ-glutamyl transferase, IU/L	5–55	107	31	23
Aspartate aminotransferase, IU/L	15–37	20	16	14
Alanine aminotransferase, IU/L	12–78	28	26	22
*NA, not applicable. †Tissues were cultured for bacteria, fungi, and mycobacteria.				

Blood cultures remained sterile. Serologic test results were negative for HIV, amebiasis, and echinococcosis, but positive for *Yersinia enterocolitica* serotype O:9 and *Brucella* sp. (Table). HUS-guided drainage of the abscess yielded thick purulent fluid. Fluid cultures were negative, but we detected *Brucella melitensis* DNA by PCR amplification and sequencing of the 16S rRNA–encoding gene. Histological findings of liver tissue were compatible with a chronic abscess. We confirmed diagnosis on 2 occasions by PCR detection of *Brucella* DNA in the liver abscess, as previously reported (*1*,*3*,*5*–*7*).The serologic profile was suggestive of chronic brucellosis combining low IgM but strong IgG *Brucella* antibody titers (*1*,*3*,*5*,*7*). However, *Brucella* serologic diagnosis is poorly specific, due to antigenic cross-reactions (e.g., *Y. enterocolitica* 0:9) and residual antibodies related to past exposure in patients from *Brucella-*endemic areas (*3*). *Brucella* cultures of blood or tissue samples and PCR on serum are usually not contributing factors in chronic focal brucellosis (*1*,*3*,*5*–*7*).

Upon diagnosis of hepatic brucellosis, we administered doxycycline plus rifampin, replacing initial empirical treatment with ceftriaxone and metronidazole for suspected pyogenic abscess. After 2 months of treatment, clinical and biologic abnormalities had regressed. CT with contrast revealed substantial reduction of the abscess, but also thrombosis of the median hepatic vein, infiltration of the gallbladder, and new lesions in segment VIII (Appendix Figure, panel B). We continued the antimicrobial therapy and surgery was recused. After 6 months of treatment, antibody titers were decreasing (Table). Three months later, positron emission tomography highlighted intense focal uptake in segments IV and VIII. Fluid from a second HUS-guided drainage was positive by *Brucella*-specific PCR test (Table). We changed the treatment to trimethoprim/sulfamethoxazole plus doxycycline for another 6 months. At completion, magnetic resonance imaging showed abscess reduction with stable central calcification. 

HB patients are difficult to manage because of lack of optimized treatment (*1*,*8*), and clinical course is the only reliable evidence in disease control (*1*,*3*,*9*).Our treatment was conservative, including 15 months of antimicrobial therapy and repeated HUS drainages. We continued treatment despite worsening radiological findings (including onset of cholecystitis and deep vein thrombosis) on the basis of early favorable clinical course and patient compliance (*1*–*3*,*8*,*9*). We monitored the improvement of radiological lesions using positron emission tomography–computed tomography and magnetic resonance imaging, both of which are sensitive for diagnosis although not assessed for monitoring, for 15 months after treatment. Few studies have described the radiological evolution over time in complicated cases (*1*–*3*,*5*,*8*), but persistence of calcifications is usual in HB (*2*,*3*,*9*).

Administration of doxycycline plus rifampin, an aminoglycoside, or both is currently recommended for brucellosis (*1*–*3*); choice and duration of treatment must be individualized to the patient’s symptomatology and treatment tolerability (*1*–*3*,*8*,*9*) and the involved tissues (*2*,*8*). Surgical resection and percutaneous drainage of the abscess are both reported as effective adjunctive therapy, with surgery indicated when evacuation of pus is ineffective or clinical symptoms persist despite drainage (*1*–*3*,*5*,*6*). A short course of an aminoglycoside could have been added at the time of radiological diagnosis of complications, as reported in other focal brucellosis with poor clinical evolution (*2*,*8*,*9*), in which a prolonged regimen (6–52 weeks) is also described (*3**,**8**,**9*). As of January 2019, after 2.5 years of treatment and follow-up care, our patient is considered cured. Relapses mainly occur within a few months of treatment completion but may occasionally occur later (*2*,*5*).

Any patient with characteristic liver abscess after traveling in brucellosis-endemic areas should undergo serologic testing for a presumptive diagnosis, and PCR testing of abscess fluid or hepatic tissue for confirmation of brucelloma. A conservative treatment combining long-term antimicrobial therapy and repeated HUS drainage may be effective. Long-term clinical comprehensive follow-up is required.

Appendix Additional information about hepatic brucellosis in a patient, Grenoble, France.
