# Supplementary material for: Hepatic Brucelloma Diagnosis and Long-Term Treatment, France
Source: Emerg Infect Dis. 2019 May;25(5):1021–3. doi: 10.3201/eid2505.180613 (PMC6478221; doi:10.3201/eid2505.180613)
Supplement: Appendix — Additional information about hepatic brucellosis in a patient, Grenoble, France. [file 18-0613-Techapp-s1.pdf]

Article DOI: https://doi.org/10.3201/eid2505.180613

Hepatic Brucelloma Diagnosis and Long-term Treatment, France

Appendix

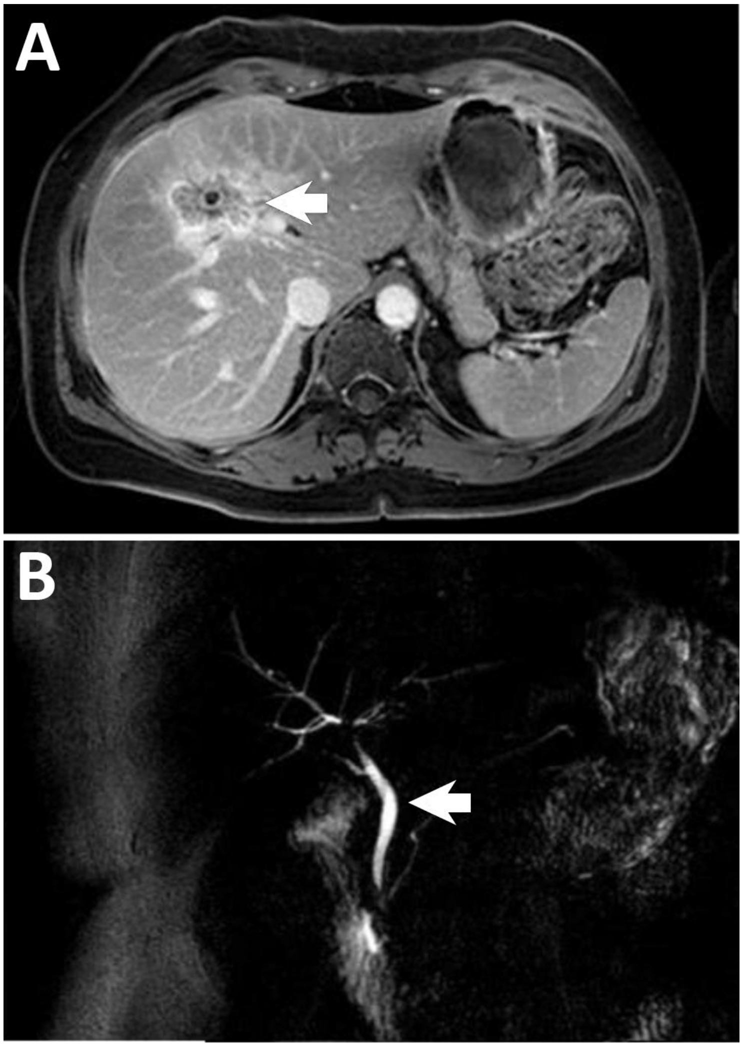

Appendix Figure. Computed tomography image showing a liver abscess in a patient with hepatic brucellosis at the time of diagnosis. Arrow indicates a hypodense lesion with a peripheral enhancement of the density and a central calcification. B) Computed tomography image of the liver of a patient with hepatic brucellosis after a 2-month treatment with doxycycline plus rifampin. Arrow indicates thrombosis of the median hepatic vein and infiltration of the gallbladder.
